# Supplementary material for: Phylogenetic Weighting Does Little to Improve the Accuracy of Evolutionary Coupling Analyses
Source: Entropy (Basel). 2019 Oct 12;21(10):1000. doi: 10.3390/e21101000 (PMC6818970; doi:10.3390/e21101000)
Supplement: Supplementary file 1 [file entropy-21-01000-s001.pdf]

# Supplementary Information for:
# Phylogenetic weighting does little to improve the accuracy of evolutionary coupling analyses

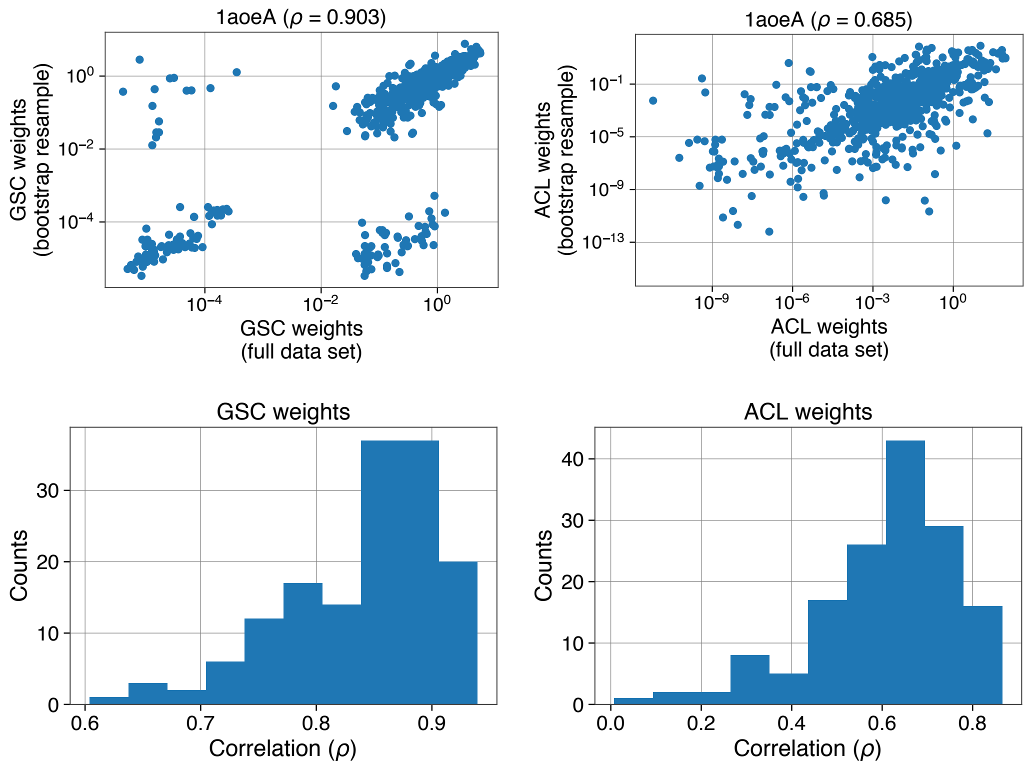


SI Fig. S1: Assessing the robustness of phylogenetic weights to errors in tree construction and rooting. We performed bootstrap re-sampling of multiple sequence alignments followed by tree construction and rooting as described in the main text. The top row shows the correlation of the resulting GSC and ACL weights for an example protein (1aoeA) compared to the weights calculated on the full alignments. The bottom row shows the correlation observed across all 150 proteins for the indicated weighting schemes.

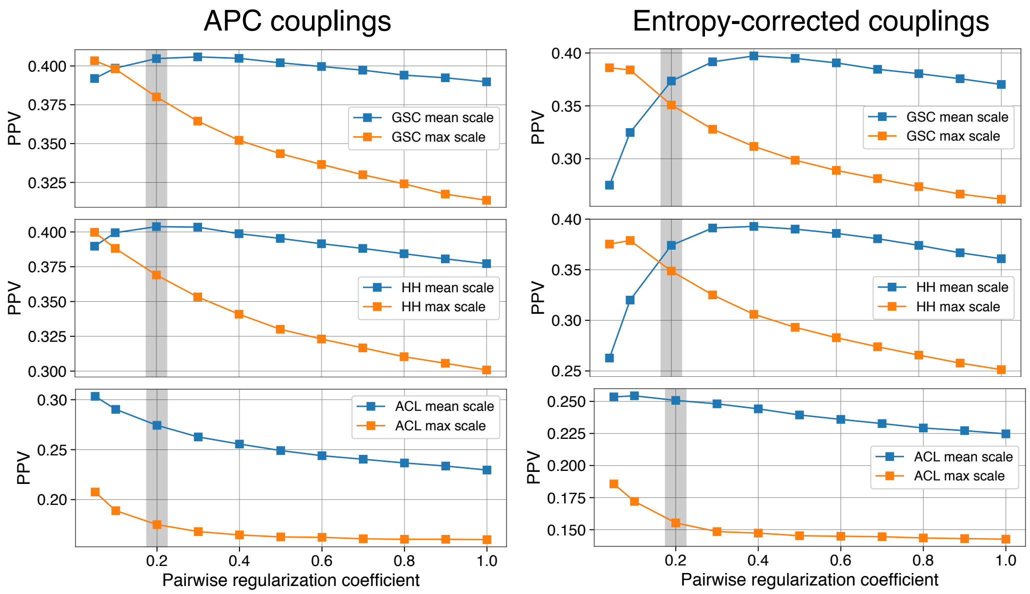


SI Fig. S2: For each of the indicated weighting methods (rows) and *post hoc* corrections (columns), we show the average (across all 150 proteins) PPVs across a range of pairwise regularization coefficients ('LFACTOR' in CCMPRED). The default parameter is highlighted by the gray vertical stripe.
